# Cutaneous metastasis of prostate carcinoma treated with radiotherapy: a case presentation

**DOI:** 10.1186/1756-0500-7-505

**Published:** 2014-08-08

**Authors:** Gabriel Mak, Melvin Chin, Najmun Nahar, Paul De Souza

**Affiliations:** Prince of Wales Hospital; Prince of Wales Clinical School, University of New South Wales, Sydney, Australia; Crown Princess Mary Cancer Centre, Westmead Hospital, Sydney, Australia; Ingham Institute, School of Medicine, University of Western Sydney, Liverpool Hospital, Sydney, Australia

**Keywords:** Prostate cancer, Metastasis, Cutaneous metastasis, Radiotherapy

## Abstract

**Background:**

Prostate cancer is a commonly diagnosed and treated malignancy, although it rarely presents with cutaneous metastases. In this case presentation, we describe the diagnosis and treatment with radiotherapy of a patient who presented with cutaneous metastases on his chest wall secondary to prostate cancer.

**Case presentation:**

In 2006, a 73-year-old Caucasian gentleman with metastatic castration resistant prostate cancer treated with mitoxantrone and prednisolone presented with cutaneous nodules on his chest wall. A punch biopsy diagnosed cutaneous metastases, with histological confirmation with positive staining for cytokeratin, PSA (prostate specific antigen) and PAP (prostatic acid phosphatise). Systemic treatment was ceased due to progressive disease; radiotherapy was used to treat these nodules with a durable clinical response. The patient died five months after initial diagnosis of cutaneous metastases.

**Conclusions:**

In this report, a rare metastatic manifestation of a common malignancy is presented. Whilst dermal metastases carries a poor prognosis from reported literature, this is the first report of radiotherapy providing a durable clinical response with relief from bleeding and pain.

## Background

Prostate cancer is the most commonly diagnosed cancer in Australia, with an estimated incidence of 18,560 in 2012 [1]. However, the skin is an uncommon site of metastasis and constitutes 5.3% of metastatic sites for all cancers [2]. In prostate cancer, cutaneous metastasis is very rare with a published incidence of 0.36% [3] - when it occurs, it usually presents as nodules in the inguinal region and anterior thigh [4]. Cutaneous metastases can also be inflammatory, fibrotic or sclerodermoid [5], and symptomatically, can be on a spectrum of being asymptomatic to being ulcerative and painful [6].

Metastasis to the chest wall is uncommon, although there is one case report published of cutaneous metastasis to the neck and upper chest [7]. The mechanism of spread is not entirely understood – direct extension, lymphatic and haematogenous spread or combination of these, have all been proposed [3, 6, 8–10].

We report herin a patient with metastatic castration resistant prostate cancer, who presented with cutaneous nodules confirmed to be metastases from his prostate cancer. This was subsequently treated by radiotherapy, with a clinically durable response. It is of interest for the rarity of its presentation, and potential treatment with radiotherapy.

## Case presentation

In 2006, a 73-year-old Caucasian man presented with nodular lesions on the left chest wall while receiving mitoxantrone and prednisolone for metastatic hormone refractory prostate cancer with skeletal metastases. At the time, mitoxantrone was the standard systemic chemotherapy for this condition, this gentleman was first diagnosed with prostate cancer 9 years previously. Past treatments included androgen deprivation, strontium, carboplatin–etoposide, radiotherapy to the chest wall for pain in the manubrium and sternum; and also with cisplatinum–phenoxidiol (as part of a phase 1 clinical trial). He initially responded well to mitoxantrone with a fall in the serum prostate specific antigen (PSA) and resolution of bone pain.

After the eighth cycle of mitoxantrone, the patient reported having skin lesions on his chest. The lesions were non-tender, pink, raised and occurred in irregular clusters just lateral to the nipple towards the mid axillary line, there were no epidermal changes. A clinical suspicion of cutaneous metastases was considered along with the differential diagnoses of a drug eruption and other dermatoses.

**Figure 1:**
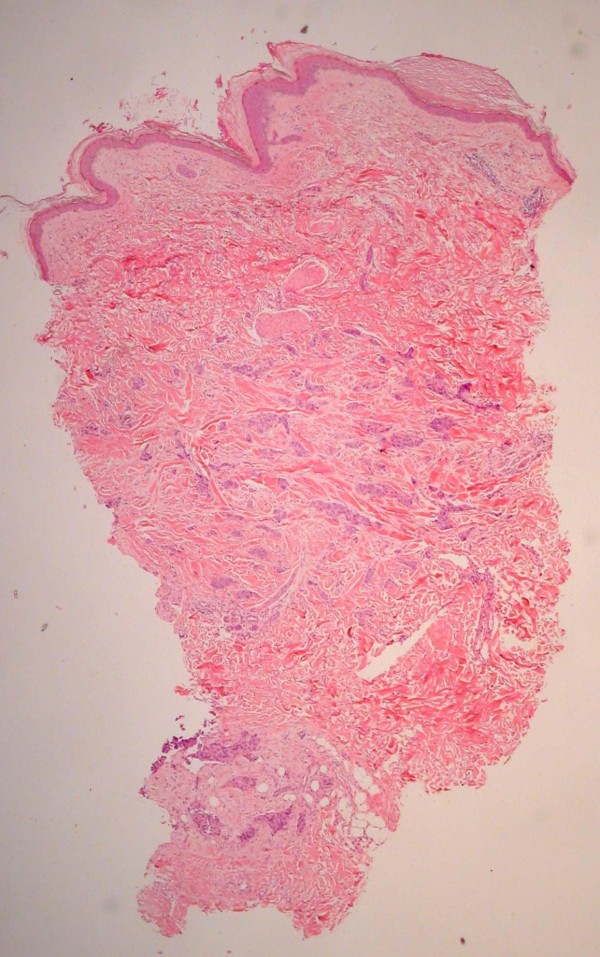
**Dermal infiltrate –** **Haematoxylin and eosin (** **H & E)** **stain.**

**Figure 2:**
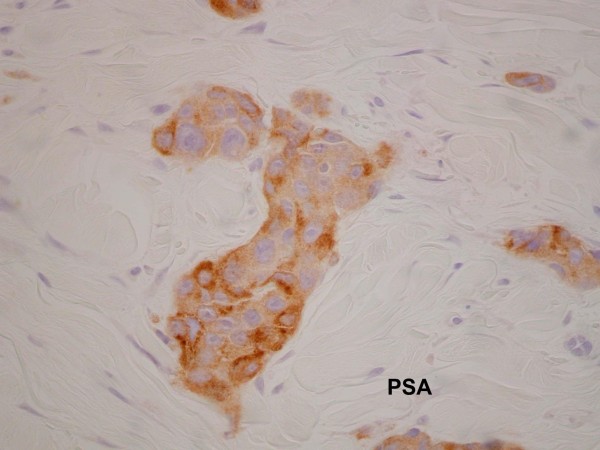
**Dermal infiltrate -** **positive for prostate specific antigen (** **PSA)** **stain.**

A punch biopsy was performed on a sample lesion. The pathology showed metastatic adenocarcinoma in the dermis that stained for cytokeratin, PSA and prostatic acid phosphatase (PAP) consistent with prostate cancer (Figures 1 and 2). He was also found to have a lower leg deep vein thrombus and was commenced on enoxaparin then warfarin.

**Figure 3:**
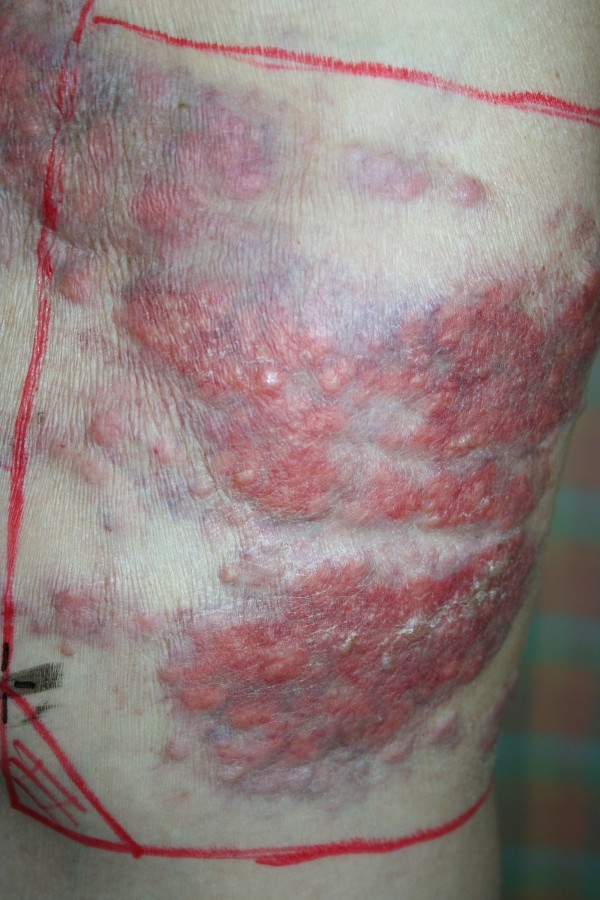
**Left lateral chest wall**, **two months after first presentation.**

**Figure 4:**
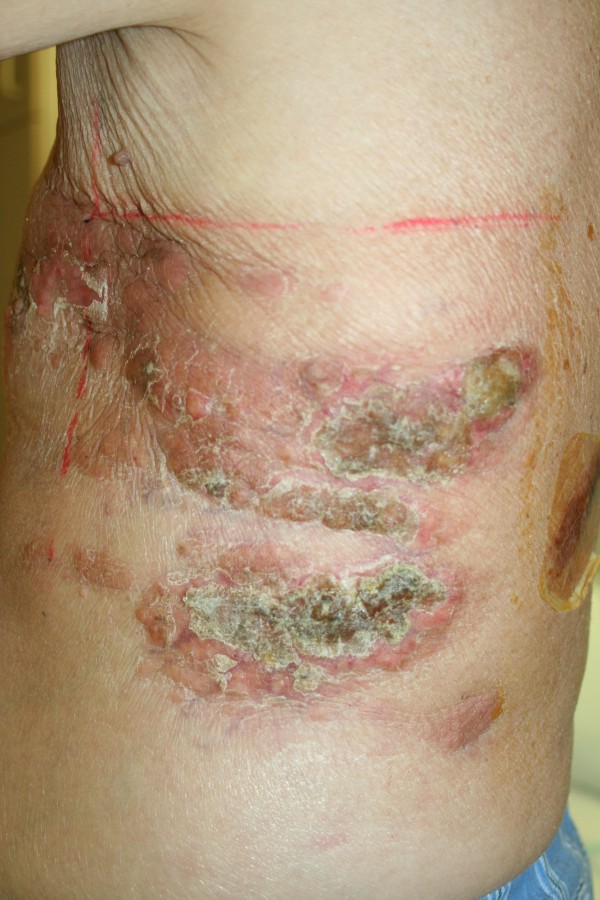
**Left lateral chest wall,** **39 days post radiotherapy treatment.**

Chemotherapy was ceased due to progressive disease, thereafter, serum PSA levels continued to rise. The PSA increase coincided with increasing extent, size and pain in the nodules on the chest wall. The lesions close to a previous sternal ulcer became friable and bled easily (Figure 3). Two months from when the lesions were first noticed, palliative radiotherapy of 18 Gy in 3 fractions using electrons was delivered to the left chest wall to good clinical effect (Figure 4).

The PSA continued to rise, however, and further symptoms developed including fatigue, leg swelling and dyspnoea. A computed tomography (CT) scan revealed bilateral pleural effusions, the larger effusion on the left was drained. The cytology of the fluid revealed adenocarcinoma cells staining with anti-cytokeratin antibody CAM 5.2, PSA and PAP consistent with metastatic disease.

The treated cutaneous lesions did not cause further problems but new lesions were found out of the field of radiotherapy treatment (Figure 4). He continued to decline and required a second pleurocentesis. At this time there were also changes on CT consistent with pulmonary lymphangitis, he subsequently died in hospital 2 weeks later.

## Conclusions

In prostate cancer, dermal involvement generally presents late in the disease and is associated with poor prognosis [3, 6]. One review of literature estimates mean survival time from diagnosis of cutaneous metastasis at being 7 months [9]. In this case, the patient had metastatic prostate cancer for 9.5 years in total and died 5 months from the initial diagnosis of the skin metastases. These occurred at failure of biochemical response to chemotherapy, and became more extensive and symptomatic as the PSA increased.

The mechanism of spread is uncertain – in our case, the finding of pleural effusions with cytology positive for prostate cancer cells in this case raises the possibility of a transmural infiltration of metastases in the chest wall. Histologically, there are multiple reported morphological patterns, and it typically shows immunohistochemistry positivity for PSA and PAP. However, expected morphology and staining may not be present in a poorly differentiated specimen [8, 11, 12].

This patient also had a significant and durable local response to radiotherapy. The symptoms of bleeding and pain completely subsided after treatment with 18 Gy in 3 fractions; although this did not lower serum PSA or stop systemic progression.

Treatment options for cutaneous metastases include local excision, intralesional chemotherapy [9] or radiotherapy. Our literature review revealed no published reports specifically for radiotherapy treatment for cutaneous metastases of prostate carcinoma. In this case, we report that radiotherapy can be a safe and effective treatment with a durable response in this rare metastatic presentation of a common tumour.

## Consent

Written informed consent was obtained from the patient, prior to his death, for publication of this case report and accompanying images. Furthermore, written informed consent from the patient’s next-of-kin was also sought post mortem, and a copy of the written consent is available for review by the Editor-in-Chief of this journal.
